# Supplementary material for: Drowning prevention challenges and opportunities: An exploratory study of perspectives of delegates from ASEAN nations
Source: PLoS One. 2024 Jun 6;19(6):e0304138. doi: 10.1371/journal.pone.0304138 (PMC11156313; doi:10.1371/journal.pone.0304138)
Supplement: Attachment — Submitted filename: Letter of response to reviewers.docx [file pone.0304138.s001.docx]

24-04-2023

Jasmin C Lawes, Ph.D.
Academic Editor
PLOS ONE

Dear Dr Lawes,

Thank you for the opportunity to revise manuscript ID PONE-D-24-10409 entitled “Drowning Prevention challenges and opportunities: Perspectives of delegates from ASEAN nations” which we submitted for consideration for publication in PLOS ONE.

We are pleased to see there is significant support for our manuscript. We have addressed each of the journal requirements and comments from the reviewers in the letter below but also in the manuscript as required using track changes function.

We believe the manuscript is further improved and hope it is now acceptable for publication in PLOSONE.

Yours sincerely,

Dr Amy Peden

a.peden@unsw.edu.au

Journal Requirements

1. Please ensure that your manuscript meets PLOS ONE's style requirements, including those for file naming. The PLOS ONE style templates can be found at

<https://journals.plos.org/plosone/s/file?id=wjVg/PLOSOne_formatting_sample_main_body.pdf> and

<https://journals.plos.org/plosone/s/file?id=ba62/PLOSOne_formatting_sample_title_authors_affiliations.pdf>

Author response: Thank you, we have checked these links and believe we comply with the style requirements and file naming conventions.

2. In this instance it seems there may be acceptable restrictions in place that prevent the public sharing of your minimal data. However, in line with our goal of ensuring long-term data availability to all interested researchers, PLOS’ Data Policy states that authors cannot be the sole named individuals responsible for ensuring data access (<http://journals.plos.org/plosone/s/data-availability#loc-acceptable-data-sharing-methods>).

Data requests to a non-author institutional point of contact, such as a data access or ethics committee, helps guarantee long term stability and availability of data. Providing interested researchers with a durable point of contact ensures data will be accessible even if an author changes email addresses, institutions, or becomes unavailable to answer requests.

Before we proceed with your manuscript, please also provide non-author contact information (phone/email/hyperlink) for a data access committee, ethics committee, or other institutional body to which data requests may be sent. If no institutional body is available to respond to requests for your minimal data, please consider if there any institutional representatives who did not collaborate in the study, and are not listed as authors on the manuscript, who would be able to hold the data and respond to external requests for data access? If so, please provide their contact information (i.e., email address). Please also provide details on how you will ensure persistent or long-term data storage and availability.

Author response: Thank you for this. We have indicated data are also available via request to the organisation Royal Life Saving Society – Australia by email (please see the revised manuscript). Data will be stored long term on the RLSSA server.

3. Please review your reference list to ensure that it is complete and correct. If you have cited papers that have been retracted, please include the rationale for doing so in the manuscript text, or remove these references and replace them with relevant current references. Any changes to the reference list should be mentioned in the rebuttal letter that accompanies your revised manuscript. If you need to cite a retracted article, indicate the article’s retracted status in the References list and also include a citation and full reference for the retraction notice.

Author response: Thank you for this feedback. We have checked the reference list and confirm it is correct and includes no retracted studies. No further action required.

**Reviewer Comments**

Reviewer #1: This study identified the barriers to implementing the United Nations General Assembly resolution on global drowning prevention, as well as opportunities for cross-seeding drowning prevention ideas and opportunities in ASEAN nations. I have some minor suggestions for further improvement.

Author response: Thank you for the time taken in conducting this review and for your support of our manuscript.

1. The authors should articulate the design of this study, e.g., the mixed method study, co-design study or other.
Author response: Thank you for this suggestion. We used surveys to gather both qualitative and quantitative data. As this area of research is really at the start in such nations, we have changed the title to refer to this as an exploratory study. Please see the revised manuscript.

2. Why the number of participants in the pre-survey was less than that in the post-survey?

Author response: Thank you for this question. Like all surveys, we suffered some attrition in our response rate between the pre and the post surveys. We have noted this in the strengths and limitations section as follows:

The data presented represented the views of a small number of invited workshop participants only, with some attrition in response rates between the pre and post surveys.

3. The affiliation, professional title or research direction should be supplemented in Table 1.
Author response: Thank you for this suggestion Unfortunately due to the small sample size we are precluded from including this information for fear of identifying individual respondents. Additionally, it should be noted that the vast majority of respondents are policy makers or practitioners and not researchers.

4. How about the reliability of self-reported data about government involvement, water safety or drowning prevention plans?

Author response: Thank you for pointing this out. We do acknowledge that surveys are prone to bias such as recall and social desirability bias. By maintaining anonymity we do hope that respondents gave their answers honestly. Nevertheless, see below for the edits we have made to the strengths and limitations section of the manuscript:

Surveys are also prone to inherent limitations including social desirability and recall bias and therefore results should be interpreted with caution.

5. The discussion and limitation sections were good.

Author response: Thank you for your support of our work, no further action required.

6. The figure was not clear.

Author response: Thank you for this feedback. We can see the figure was not uploaded at its highest resolution, nor was it uploaded with its figure caption (see it in situ in the manuscript – line 208). We have ensured the figure is uploaded in its highest resolution, and we have also further clarified the figure title as follows:

**Figure 1: Distribution of Government ministries reported to be involved in drowning prevention across selected ASEAN countries**

Reviewer #2: Thank you for this scholarship in a space of great need.
Author response: Thank you for the time you took to perform the review and the support of our manuscript.

Title: no comment
Author response: Thank you, no further changes required.

Abstract:
Line 41 consider removing the word "grouping"
Author response: Thank you for this suggestion, we agree it improves readability. Please see the manuscript to see this deletion.

Line 50 consider removing the word "thus"
Author response: Thank you for this suggestion we have edited this line as suggested. Please see the revised manuscript.

Introduction: well done
Author response: Thank you, no further changes required.

Methods:
a) As the level of scholarship in drowning prevention elevates, consenting by completion or submission of a questionnaire will become non-ideal. When these research methods are combined (in this case, the research consenting and the completion/submitting the questionnaire), it becomes unclear if the non-completion is due to non-consent, conflicts of interest or just did not complete it.

Author response: Thank you for this feedback. Although this method was pre-approved prior to the project commencing by the University Human Research Ethics Committee, we do agree it makes it hard to determine the reasons for non-completion of the survey. We have added this to the strengths and limitations section as follows:

Consent to participate was recorded via the return of the questionnaire, which did not allow for determination of reasons for non-completion. Although reasons for non-completion aren’t known, given the desire for a common strategy on drowning prevention across the region, the lack of participation from representatives of Cambodia and Myanmar in the workshop and surveys likely indicate a need to foster drowning prevention efforts in these countries.

b) If I understand correctly - there were 30 people sent a pre-workshop questionnaire and that same 30 people were sent the post-workshop questionnaire. If this is not the correct interpretation, please provide clarification.

Author response: That is correct, no further action required.

c) If the demographic information was attached to both survey submissions - due to the small number of participants from each country the data manager is likely to be able to identify the responders by country. I understand the statement in line 165 in Data Analysis about "combining to preserve anonymity" but it was unlikely that the data was anonymous to the data manager. I would agree that the data responses are anonymous to the reader of the paper. Consider making a clarifying comment about the challenges of anonymous response in a survey of this small cohort of country based participants.
Author response: Thank you for this. Yes you are correct, the data were presented in an anonymous fashion for the reader and as per ethical guidelines. We have added the following in the data analysis section at line 166 to clarify: As drowning is an emerging area of work within the region, the socio-demographic information of respondents across both surveys were combined to preserve anonymity when displayed in the manuscript.

Further, we have included a statement in the strengths and limitations section of the manuscript regarding this challenge as follows:

The small number of country-based participants made maintaining anonymity in presentation of results challenging.

Results:
a) Line 176/177 - consider sharing the more full survey response data - if I have understood the method correctly- 30 participants got sent a pre and post workshop survey = 60 surveys sent. 9 participants responded to the pre-workshop survey = 9/30 = 30% response rate. 13 participants responded to the post-workshop survey = 13/30 = 43% response rate. A total of 22 responses were received from 60 surveys sent out = 37% response rate. This may not be a weakness of the study if the method was to get a response from the each country (that did happen assuming that 2 nations did not attend).

Author response: Thank you for these queries. We have clarified the participation and response rates, as well as country representativeness in new text added to the results section as follows:

In total, there were 30 people invited to complete the surveys (15 for the pre and post surveys respectively). A combined total of 22 responses were received, an overall response rate of 73% (60% pre survey and 87% for the post survey). Responses were received from all countries who participated in the workshop aside from Malaysia. All ASEAN nations were represented at the workshop aside from Cambodia and Myanmar. Socio-demographic information of respondents can be found in Table 1.

b) The 22 responses included 2 or more participant from each of 8 of the 10 ASEAN nations.
Author response: Thank you for this comment. See above for the clarification re country participation in the survey and workshop.

c) A comment on the non-responses may be helpful - Did some countries delegate 2 participants to respond on behalf of the country (government and NGO) and therefore the non-responders were redundancies??
Author response: Thank you for this suggestion. As noted prior we don’t know the reasons for non-response, nor did we indicate a need to limit to 2 responses per country. Based on reviewer feedback we have indicated that there are limitations around the survey design and capturing reasons for non-response. Please see the revised manuscript.

Current Activities: well done
Author response: Thank you, no further action required.

Government Involvement:
Line 200 - consider adding heath to this list in the first paragraph.
Author response: Thank you. We have added health to this sentence. Please see the revised manuscript.

Lone 201 - remove the comma, after ministry,
Author response: Thank you for this suggestion. We have removed the comma as suggested.

Line 203-206 - consider clarifying reworking to indicate that in Lao PDR, where drowning is an emerging issue, the Ministry of Health is identified as the aspirational lead agency, rescue volunteer agencies are active in the provision of interventions currently.
Author response: Thank you for this suggestion. We have revised the sentence as follows:

In Lao PDR, where drowning is an emerging issue, although Ministry of Health was identified as the aspirational lead government agency, in reality rescue volunteers are the group responsible for drowning prevention efforts to date in the country (Figure 1).

Figure 1 - I presume is the nations flag chart at the end of the paper that is not labeled as Figure 1. I found this chart helpful. The words in the circles that represent government ministries are a bit unclear in graphic. I found it interesting that there are 7/10 ASEAN nations on this chart and the one workshop participant missing is Malaysia who were workshop hosts and survey responders - the participants from Malaysia may not have identified the government engagement?? Water Safety or drowning prevention plans:
Author response: Thank you for this feedback. The caption for the figure is embedded within the manuscript as PlosONE requests, while the figure itself is at the end of the manuscript. We have revised the figure title to be more clear on the content of the figure. We have also checked the figure file to ensure it is as high quality as possible. The revised text at the beginning of the results further clarifies Malaysia’s non-involvement in the survey despite being the workshop host country.

Drowning Data:
At line 241 there is a transition from drowning data to challenges to drowning prevention - consider a new subheading at this line; Challenges to Drowning Prevention:
Author response: Thank you for this suggestion. We have added a subheading entitled ‘Challenges in Preventing Drowning’. Please see the revised manuscript.

Discussion:
Line 258 - consider the words "has resulted" instead of "necessitates"
Author response: Thank you. We have made the change as suggested. Please see the revised manuscript.

Line 261 - consider removing the word "However" and starting with As a multi-sectoral –

Author response: Thank you for this suggestion. The change has been made as suggested. Please see the revised manuscript.

Strengths and Limitations:
Consider a comment on the wide scope or degree of engagement in drowning within the survey and workshop participant group. Some may have been assigned their participation and others have active action for a long time and have participated in the growth of global drowning prevention awareness and activities.
Author response: Thank you for this suggestion. We have added the following to the strengths and limitations section:

The views of respondents indicate a diverse degree of engagement in drowning prevention ranging from emerging activities through to many years of action on the issue.

Conclusion: well done

Author response: Thank you for your support of our research.

Thank you for this opportunity to review your good work.

Author response: Thank you for your time in conducting this review and your support of our research.
